# A Comparative Study of Interaction of Tetracycline with Several Proteins Using Time Resolved Anisotropy, Phosphorescence, Docking and FRET

**DOI:** 10.1371/journal.pone.0060940

**Published:** 2013-04-11

**Authors:** Manini Mukherjee, Pinki Saha Sardar, Shyamal Kr. Ghorai, Swarna Kamal Samanta, Atanu Singha Roy, Swagata Dasgupta, Sanjib Ghosh

**Affiliations:** 1 Department of Chemistry, Presidency University, Kolkata, India; 2 Department of Chemistry, Indian Institute of Technology, Kharagpur, India; University of Leeds, United Kingdom

## Abstract

A comparative study of the interaction of an antibiotic Tetracycline hydrochloride (TC) with two albumins, Human serum albumin (HSA) and Bovine serum albumin (BSA) along with *Escherichia Coli* Alkaline Phosphatase (AP) has been presented exploiting the enhanced emission and anisotropy of the bound drug. The association constant at 298 K is found to be two orders of magnitude lower in BSA/HSA compared to that in AP with number of binding site being one in each case. Fluorescence resonance energy transfer (FRET) and molecular docking studies have been employed for the systems containing HSA and BSA to find out the particular tryptophan (Trp) residue and the other residues in the proteins involved in the binding process. Rotational correlation time (θ_c_) of the bound TC obtained from time resolved anisotropy of TC in all the protein-TC complexes has been compared to understand the binding mechanism. Low temperature (77 K) phosphorescence (LTP) spectra of Trp residues in the free proteins (HSA/BSA) and in the complexes of HSA/BSA have been used to specify the role of Trp residues in FRET and in the binding process. The results have been compared with those obtained for the complex of AP with TC. The photophysical behaviour (viz., emission maximum, quantum yield, lifetime and θ_c_) of TC in various protic and aprotic polar solvents has been determined to address the nature of the microenvironment of TC in the protein-drug complexes.

## Introduction

The interaction of a protein with a drug or a toxic molecule is usually studied by steady state and time-resolved quenching of Trp emission of proteins. [1]–[15] In many cases the ligand (the drug or the toxic molecule) which is usually a poor emitter in aqueous medium exhibits enhanced emission due to interaction with protein. In such cases, exploiting the ligand emission is advantageous to study the interaction, since Trp emission in protein may be partly quenched intrinsically by nearby residues due to subtle change of conformation around the Trp residues in the protein-ligand complex.

Steady state fluorescence, using selective excitation, along with steady state and time-resolved anisotropy studies of Trp residues in proteins and in the complexes of proteins with other molecules are widely used to identify structural perturbation and other dynamical information. [1] The other molecules include nucleic acid, protein and suitable ligand, acting as drug or toxic molecule. Although the fluorescence spectra of Trp residues in proteins are usually broad, the position of the fluorescence maxima provides an overall signature of Trp environment in proteins or in the complexes of proteins. However, it is not possible to find the contribution of individual Trp in the fluorescence spectra of multi Trp proteins. The fluorescence lifetime even in a protein having single Trp residue exhibits multiexponential decay. Thus it is also difficult to assign the lifetime value to a particular Trp residue in the free protein as well as in the complex of protein with other molecules. [1].

The phosphorescence of Trp residues in proteins at 77 K in a suitable cryosolvent forming a glassy matrix, on the other hand, always appears as structured spectra with a definite (0,0) band, characteristic of the Trp environment. [16]–[38] The position and the width of the (0,0) band along with the overall structural features of the spectra provide a definitive information regarding the polarity, nature of the solvent exposure, the hydrophobicity and the homogeneity of the immediate environment of the Trp residue. The narrowness of the (0,0) bands of phosphorescence spectra at 77 K is attributed to the smaller excited state dipole moment of the lowest (π-π*) triplet state of Trp. [39] The blue-shifted phosphorescence (0,0) band, typical of free Trp in a polar solvent represents a Trp residue in polar environment with greater solvent exposure. The red-shifted (0,0) phosphorescence band is characteristic of a Trp residue located in a buried polarizable environment with less solvent exposure. [9], [17], [39] Specific interactions with polar residues, however, could result in blue-shifted origins for buried Trp residues. [17], [40] In several cases LTP spectra of multi Trp proteins exhibit more than one (0,0) bands corresponding to different Trp residues present in the protein. [17]–[38] This occurs if the Trp residues have widely different local environment and the intramolecular singlet↔singlet nonradiative energy transfer (ET) between the different Trp residues is inherently prevented due to special architecture of the protein. The tyrosine (Tyr) residues in proteins also exhibit a broad phosphorescence spectra within 350–450 nm region with λ_exc_ = 280 nm provided Tyr-Trp intramolecular energy transfer within the protein is prevented. [41].

Thus LTP of protein along with conventional steady-state and time-resolved fluorescence spectra can provide a meaningful understanding of protein-protein interaction, [34] protein-nucleic acid interaction [42] and protein-ligand [13], [20], [43] interactions.

**Figure 1:**
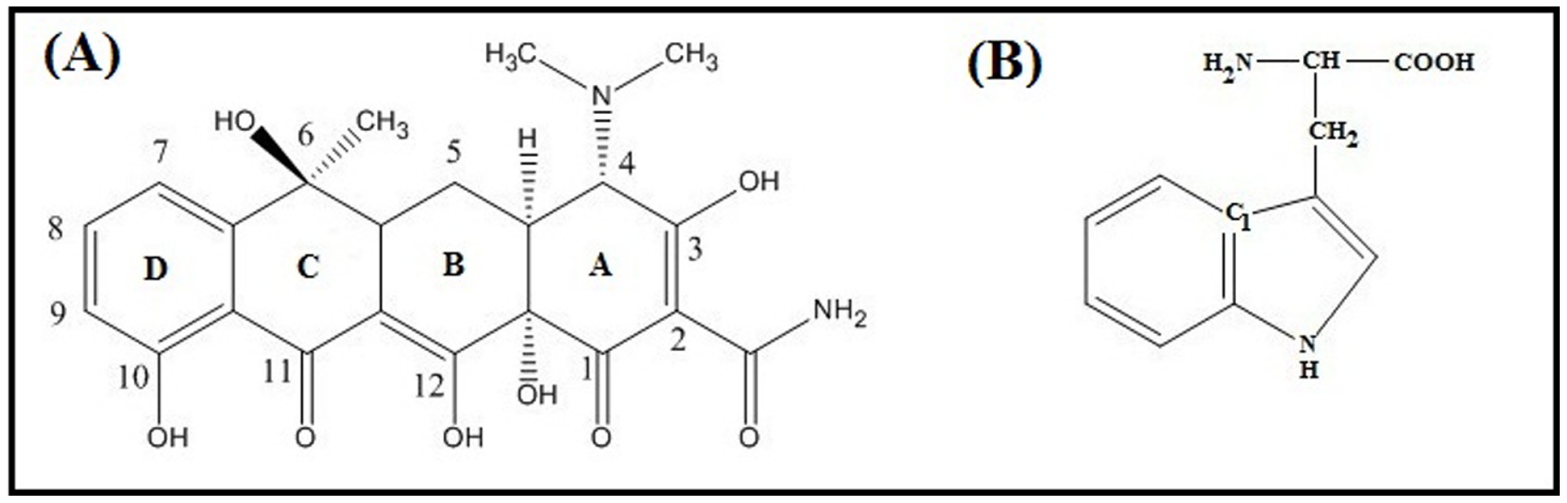
Structure of (A) Tetracycline and (B)Tryptophan residue.

In this work we report the interaction of TC with serum albumins (BSA/HSA) along with a comparative study with AP-TC interaction. [20] The basic molecular structure of TC, discovered in 1948 as natural fermentation products of a soil bacterium, Streptomyces aureofaciens is shown in Figure 1. The ring structure of TC is surrounded by upper and lower peripheral zones where various chemical functional groups can be attached.[44]–[46] Apart from its antibiotic activity, it also affects inflammation, immunomodulation, cell proliferation and angiogenesis. [47]–[49].

Serum albumins, the most abundant protein in the blood stream, account for about 60% of the total plasma protein. The most important physiological function of these proteins is to bind and transport several exogenous and endogenous molecules like fatty acids, nutrients, steroids, and sparingly soluble drugs. HSA is a globular protein consisting of 585 amino acids and is cross linked by seventeen disulfide bonds. It is considered to have three specific domains, I, II, and III, each of which consists of two subdomains a and b possessing common structural motifs. [50] HSA contains a single tryptophan residue at 214.

A comparative study of the amino acid sequences of BSA and HSA by Brown shows that they have similar general structural features, the difference in sequence being generally conservative. [51]The BSA molecule is made up of 3 homologous domains which are divided into 9 loops by 17 disulfide bonds. BSA has two tryptophan residues, Trp 134 in the first domain and Trp 213 in the second domain. [52] BSA and HSA have 20 and 18 tyrosine (Tyr) residues, respectively.

The functional form of the metalloenzyme AP consists of a homo dimer with an appropriate mass of 94 KDa. [53]–[54]The three metal ions (two Zn^2+^ ions and one Mg^2+^ ion) in the three active sites of AP form a catalytic metal triad similar to that of the phospholipase C from Bacillus cereus [55] and P1 nuclease from Penicillium citrinum [56]. Each monomer of AP contains three Trp residues at 109, 220 and 268. The three Trp residues have been well characterised. The quasi-crystalline environment of the buried Trp 109 is evidenced by the narrow bandwidth of the optically detected magnetic resonance (ODMR) transition in zero magnetic field.[18]–[19] The Trp 220 is found to be partially exposed. [18]–[19] The emission of the Trp 268 is intrinsically quenched due to its close proximity to Cys 286-Cys336. [18]–[19].

In this report we emphasize the following to unravel the different features of interaction of TC with the three proteins:

- Evaluation of the binding constant from the enhanced emission of bound TC and anisotropy of the bound TC.
- FRET and the rate constants of energy transfer (ET) using time resolved emission of the donor Trp in proteins and also the emission of TC, acting as acceptor.
- Time resolved anisotropy monitoring the emission of bound TC to find the rigidity of the microenvironment of TC imposed by binding.
- Molecular docking studies of protein-TC complexes to find the residues involved in the binding in each case and to identify Trp residue involved in FRET.
- Phosphorescence study at 77 K to find the role of Trp/Tyr residues in FRET.
- Emission and anisotropy features of TC in various protic and aprotic solvents to address the nature of the microenvironment and the mechanism of the binding of TC in the protein-TC complexes.

## Experimental Procedures

### 2.1 Materials

The tetracycline hydrochloride (TC) and serum albumins (HSA and BSA) were purchased from Sigma–Aldrich, USA. All the solvents used were of spectral grade and further dried by standard procedures. Phosphate buffer of pH 7 was prepared in triply distilled water and used for making experimental solutions.

### 2.2 Steady State Measurements

UV-Vis absorption spectra were recorded on a Hitachi U-3210 spectrophotometer at 298 K. The steady state emission measurements were carried out using a Hitachi Model F-4010 spectrofluorimeter equipped with a 150-W xenon lamp, at 298 K using a stopper cell of 1 cm path length. Fluorescence quantum yield (φ) was determined in each case by comparing the corrected emission spectrum of the samples with that of quinine sulfate in 0.1 N H_2_SO_4_ (φ_D_ = 0.54) [57] considering the total area under the emission curve.

The measurement of steady state anisotropy was performed in Hitachi Model F-7000 spectrofluorimeter with manual Glen Thompson polarizer. The steady state anisotropy (r) is defined by
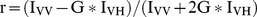
(1)where I_VV_ and I_VH_ are the intensities obtained with the excitation polarizer orientated vertically and the emission polarizer oriented vertically and horizontally, respectively. The G factor is defined as
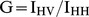
(2)‘I’ terms refer to the parameters similar to those mentioned above for the horizontal position of the excitation polarizer and vertical and horizontal position of the emission polarizer, respectively.

Steady state anisotropy measurement at room temperature was done using F-7000 Hitachi spectrofluorimeter. [58].

### 2.3 Low Temperature Phosphorescence Measurement

Emission studies at 77 K were made using a Dewar system having a 5 mm o.d. quartz tube. The freezing of the samples at 77 K were done at the same rate for all the samples. Phosphorescence was measured in a Hitachi F-7000 spectrofluorimeter equipped with phosphorescence accessories. All the samples were made in 40% EG-water for measurements at 77 K. The samples were excited at different wavelengths (280 nm and 295 nm) using a 10 nm band pass, and the emission band pass was 1 nm.

### 2.4 Time Resolved Emission Measurements

Singlet state lifetime was measured by Time Master fluorimeter from Photon Technology International (PTI,USA).The system consists of a pulsed laser driver of a PDL series i.e, PDL-800-B (from Picoquant, Germany) with interchangeable sub nano second pulsed LEDs and pico-diode lasers (Picoquant, Germany) with a TCSPC set up (PTI,USA). The software Felix 32 controls all acquisition modes and data analysis of the Time Master system. [59] Decay measurement using ‘magic angle’ detection with an emission polarizer set at 55^o^ were carried out and no detectable difference in the fitted τ values with those obtained from normal decay measurements were observed. The sample of 25 µM TC with different concentration of HSA and BSA were excited using PLS-370 (Pulse Width-600 ps) and diode laser LDH-405(Pulse Width-70 ps) respectively at a repetition frequency 10 MHz. Instrument response function (IRF) were measured at the respective excitation wavelengths, namely, 370 and 405 nm using slits with a band pass of 3 nm using Ludox as the scatterer. The decay of sample was analyzed by nonlinear iterative fitting procedure based on the Marquardt algorithm. Deconvolution technique used can determine the lifetime up to 200 ps with sub nano second pulsed LEDs and 100 ps with diode lasers. Intensity decay curves were fitted as a sum of exponential terms: [1].



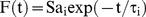
(3)where α_i_ represents the pre-exponential factor to the time resolved decay of the component with a lifetime τ_i_. The decay parameters were recovered using a nonlinear iterative fitting procedure based on the Marquardt algorithm. The quality of fit has been assessed over the entire decay, including the rising edge, and tested with a plot of weighted residuals and other statistical parameters, e.g., the reduced χ^2^ ratio and the Durbin–Watson (DW) parameters. [59] Mean lifetime<τ>for biexponential decays were calculated using the equation: [59]



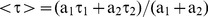
(4)Anisotropy decay measurement was also carried out in Time Master Fluorimeter (PTI, USA) using PLS-370 and motorized Glen Thompson polarizer. The anisotropy, r(t) is defined as
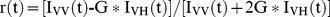
(5)where I(t) terms are defined as intensity decay of emission of TC with excitation polarizer orientated vertically and the emission polarizer oriented vertically and horizontally, respectively:
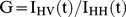
(6)where, G is the correction term for the relative throughput of each polarization through the emission optics. The entire data analysis was done with the software Felix 32 which analyses the raw data IVV and IVH simultaneously by global multi-exponential program and then the deconvolved curves (IDVV and IDVH) are used to construct r(t) [59] and from the fitted curve the correlation time (θC) can be recovered.

### 2.5 Docking Studies

The crystal structure of HSA (PDB entry 1AO6) and BSA (PDB entry 4F5S) were downloaded from the Protein Data Bank. [60] The three-dimensional structure of the tetracycline molecule was obtained from Sybyl 6.92 (Tripos Inc., St. Louis, USA) and the energy-minimized structure was achieved using a Tripos force field and Gasteiger-Hückel charges with a gradient of 0.005 kcal/mol. The dielectric constant, number of iteration, maximum displacement, minimum energy change, simplex threshold and simplex iteration were set to 1.0, 1000, 0.01, 0.05, 1000 and 20 respectively. The FlexX single molecule docking software was used to dock tetracycline with chain A of alkaline phosphatase. The ranking of the generated solutions was obtained from a scoring function as mentioned by Rarey et al. [61] PyMol was used to visualize the docked conformation and to measure the distances between the ligand and the protein. [62].

### 2.6 Accessible Surface Area Calculations

The ASA (accessible surface area) of HSA and BSA (free) and the docked complexes with tetracycline were calculated using NACCESS. [63] The best docked structure according to the minimum energy was chosen to determine the ASA of the docked proteins. The change in accessible surface area for residue *i* was calculated using the following equation.
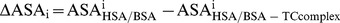



A residue was considered to be involved in the binding if it lost more than 10 Å^2^ ASA when going from the free state to the complexed state. PEARLS (Program of Energetic Analysis of Receptor Ligand System) was used to estimate the energetics of the protein-ligand interaction. [64].

## Results and Discussion

### 3.1 Steady State and Time Resolved Emission of TC in the Complexes with Proteins

**Figure 2:**
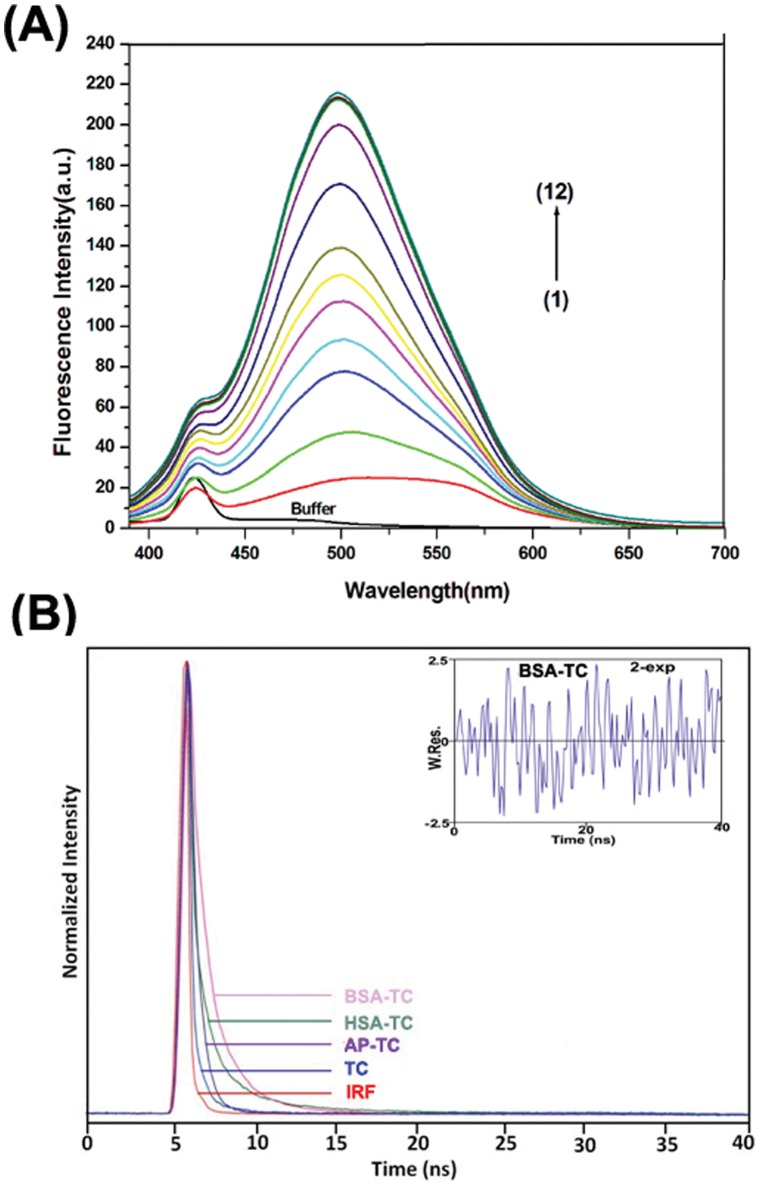
Steady State and Time Resolved Emission of TC in the Complexes with Proteins. (**A**)Fluorescence spectra of TC (25 µM) at 298 K with varying concentration of BSA; curves (1–12) represents 0 µM,2.5 µM,5.0 µM,7.5 µM,10.0 µM,12.5 µM 15.0 µM,17.5 µM,20.0 µM, 25.0 µM,40.0 µM,60.0 µM BSA, respectively; λ_exc_ = 370 nm; excitation and emission band pass = 10 nm and 5 nm respectively. (**B**) Fluorescence decay of TC (25 µM) at 298 K in aqueous buffer (pH 7), 25 µM BSA, 25 µM HSA, 25 µM AP; λexc = 370 nm; excitation and emission bandpass = 10 nm each.

**Figure 3:**
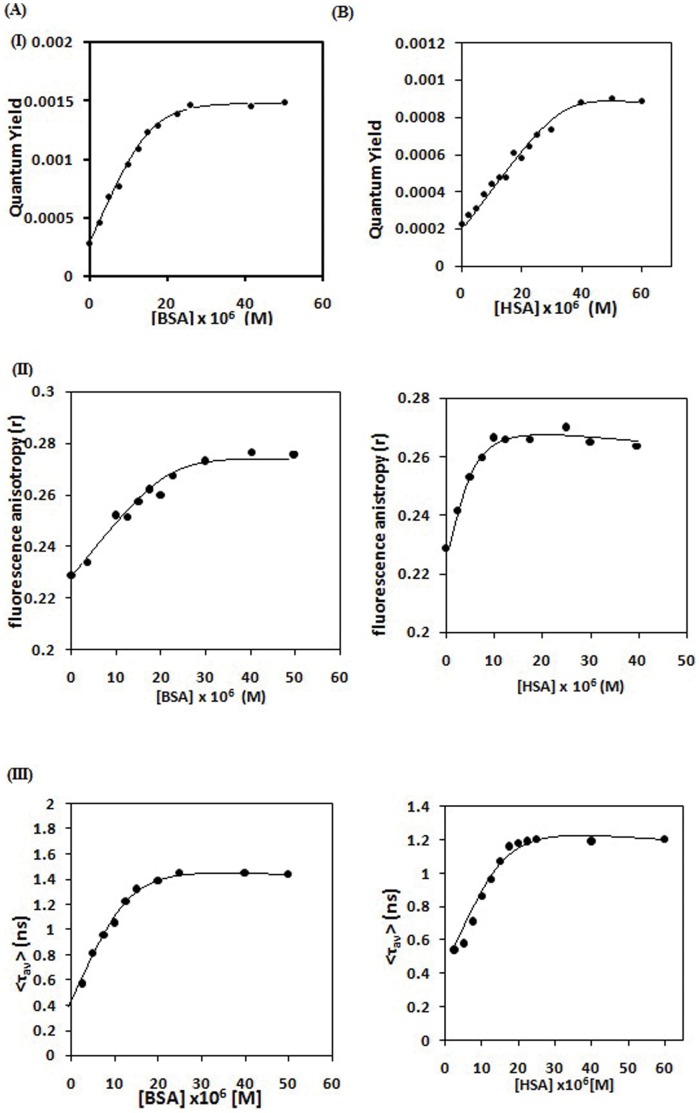
Variation of (I) fluorescence quantum yield (φ). (II) fluorescence anisotropy (r); (III) singlet state average lifetime (τ) of TC (25 µM) in aqueous buffer with increasing concentration of serum albumins. (A) for BSA, (B) for HSA.

The quantum yield of the free TC in aqueous buffer is found to be very small (Figure 2(A) Table 1). Figure 2(A) represents the emission spectra of TC (25 µM) as a function of increasing concentration of BSA using λ_exc_ = 370 nm. There has been a steady increase in the emission intensity of TC in the presence of serum albumins. The maximum of the drug emission is shifted towards blue from ∼515.0 nm in the free TC to 498.4 nm in the complex with increasing protein concentration. The similar behaviour is also observed for HSA-TC system. On comparison it has been observed that φ_HSA-TC_< φ_BSA-TC_< φ_AP-TC_. [20] The lifetime of the probe TC has been also measured as a function of added concentration of serum albumins monitoring the enhanced emission maxima in each case using λ_exc_ = 370 nm and also λ_exc_ = 405 nm (Table 1). The decays were found to fit with two components with the fitting parameter χ^2^ close to 1 in each case demonstrating the quality of fit. The representative decay profiles for the three complexes of proteins with TC are presented in Figure 2B. The average lifetime increases from 0.37 ns in free TC in aqueous buffer to 1.2 ns and 1.4 ns in HSA-TC and BSA-TC complexes respectively using both the excitations at 370 nm and 405 nm (Table 1, Figure 3).

**Table 1:** Singlet State Lifetime and Quantum Yield of TC (25 µM) as a Function of Added Concentration of Serum Albumins with λ_ex_ = 370 nm at 298 K.

System	λ_max_ (nm)	Quantum Yield (φ_f_)×10^3^	Singlet State Lifetime			
			τ_1_	τ_2_	τ_av_ (ns)	χ^2^
Free TC (25 µM)	Broad ∼515.0	0.26	1.60(5.1%)	0.30(94.9%)	0.37	1.17
+2.5 µM BSA	506.4	0.46	1.90(7.4% )	0.46(92.6%)	0.57	0.99
+5.0 µM BSA	502.8	0.68	1.99(13.6%)	0.63(86.4%)	0.81	1.10
+10.0 µM BSA	501.4	0.95	2.11(20.0%)	0.76(80.0%)	1.03	1.01
+15.0 µM BSA	500.6	1.23	2.52(21.5%)	0.99(78.5%)	1.32	1.00
+20.0 µM BSA	500.2	1.28	2.6(23.6%)	1.02(76.4%)	1.39	1.15
+25.0 µM BSA	498.4	1.55	2.62(22.8%)	1.11(77.2%)	1.4	0.99
+2.5 µM HSA	496.0	0.28	1.95(3.0%)	0.49(97.0%)	0.54	1.06
+5.0 µM HSA	492.8	0.31	2.18(4.1%)	0.51(95.8%)	0.58	1.18
+10.0 µM HSA	490.0	0.44	2.72(7.7%)	0.69(92.3%)	0.85	1.01
+15.0 µM HSA	489.8	0.48	3.04(9.9%)	0.87(90.1%)	1.07	0.99
+20.0 µM HSA	489.8	0.58	3.05(11.0%)	0.95(89.0%)	1.18	1.2
+25.0 µM HSA	489.8	0.70	3.22(11.2%)	0.96(88.8%)	1.20	1.1

### 3.2 Binding Constant and Number of Binding Sites

#### 3.2.1 From Steady State Emission and Anisotropy of Bound TC

**Figure 4:**
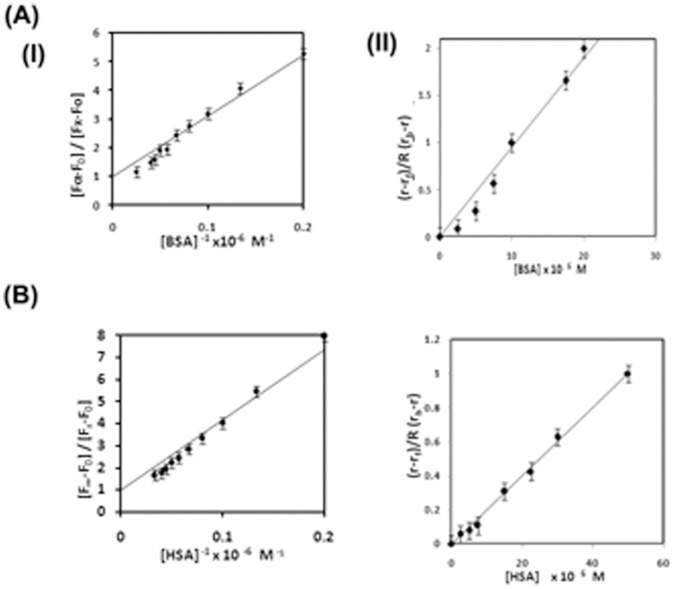
The plot of (A) (I) [F_∞_ − F_0_]/[F_x_ − F_0_] against [BSA]^−1^(II) (r−r_f_)/R(r_b_−r) against [BSA]; (B) (I) and (II) similar plot for HSA.

Although the binding constants of protein-drug complexes are usually determined from quenching of Trp fluorescence, methods exploiting the drug emission is always desirable. It is to be noted that Trp emission in protein could be perturbed not only due to bound drug molecule, but also by nearby residues of Trp due to a subtle change of conformation around Trp in the protein-drug complex. Thus we determined the binding constant from the enhanced fluorescence intensity of TC as a function of added protein concentration employing modified Benesi–Hilderbrand equation [65].


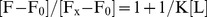
(7)

where, F_0_, F_x_, and F_∞_ are the fluorescence intensity of the enhanced emission of TC considered in the absence of protein, at an intermediate protein concentration, and at a concentration of complete interaction, respectively. K is the binding constant and [L] is the concentration of added protein (HSA and BSA). A plot of [F_∞_−F_0_]/[F_x_−F_0_] against the [L]^−1^ provides the binding constant (K) from the slope. Such a plot for the complex of TC with serum albumins is found to be linear (Figure 4) and the binding constants are given in Table 2.

**Table 2:** Binding Constant (K_b_) and Free Energy Change (ΔG) Associated with Binding Following Different Methods at 298 K.

System	Method	Binding Constant (M^−1^)	Binding Site (n)	Free Energy(Kcal/mol)	
				Experimental	Theoretical
HSA-TC	Modified Bensi-Hildebrand	3.14×10^4^	–	−6.17	−6.84
	Steady State Fluorescence anisotropy	4.49×10^4^	–		
	Steady State Fluorescence Quenching	4.94×10^4^ 5.38×10^4a^	1		
BSA-TC	Modified Bensi-Hildebrand	4.72×10^4^	–	−6.55	−7.38
	Steady State Fluorescence anisotropy	5.97×10^4^	–		
	Steady State Fluorescence Quenching	6.2×10^4^ 7.21×10^4a^	1		
AP-TC^b^	Modified Bensi-Hildebrand	6.8×10^6^	–	−9.05	−8.49
	Steady-state fluorescence anisotropy	1.9×10^6^	–		
	Steady State Fluorescence Quenching	3.9×10^6^ at 298 K	1		

a Ref [66].

b Ref [20].

We have also measured steady state anisotropy (r) of TC in the complexes at emission maxima as a function of concentration of proteins. (Figure 3) The data has been analyzed using the equation (8)
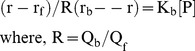
(8)


where, Q_f_ and Q_b_ are the quantum yield of the free TC and the protein bound to drug, respectively, r_f_ and r_b_ are the anisotropy observed for free TC and after complete complex formation with protein. A linear plot of (r−r_f_ )/R(r_b_–r) vs [P] provides the binding constant (Figure 4, Table 2).

#### 3.2.2 From Steady State and Time Resolved Quenching of Trp Emission

The fluorescence quenching of proteins due to the addition of TC using λ_exc_ = 290 nm was analyzed according to the following general equation: [31].
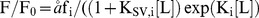
(9)where, F and F_0_ are the corrected fluorescence intensities in the presence and in the absence of drug TC, respectively, f_i_ is the fractional intensity corresponding to quenching site component i. K_sv_,_i_ is the dynamic quenching constant for the component i, K_i_ is the static quenching constant for component i, and [L] is the total quencher concentration. [31] Inclusion of the exp (K_i_[L]) term in the above equation applies in cases where there is an upward curvature in a Stern-Volmer (F_0_/F vs [L]) plot, due to static quenching. The fluorescence quenching data for the protein-drug complex was fitted to equation 9 (taking i = 1 and f_i = _1 for 1∶1 complex and assuming single site quenching) with inclusion of the static quenching term. The values of the binding constant which are in good agreement with the values obtained by other authors [66]–[67], are given in Table 2.

The binding constants determined from the steady state anisotropy data and Benesi–Hilderbrand equation monitoring the enhanced emission of the bound drug are compared with that obtained from the fluorescence quenching monitoring Trp emission of the serum albumins (Table 2). [66]–[67] The binding constants are found to be two orders of magnitude smaller than that obtained for AP-TC complexes. [20].

### 3.3 Molecular Docking Studies: Nature of Different Residues Involved in the Interaction

Molecular docking studies were performed to support the experimental data and provide some insight into the protein-drug interactions. Calculations of the ligand–receptor interaction energy from docking studies have been obtained using PEARLS.

For all three protein-TC complexes, the lowest score was taken in a FlexX run that corresponded to the best docking pose. This docked pose refers to lowest binding free energy conformation (best rank conformation) of a protein-ligand complex. This also met the criteria set for at least two consecutive minimum score values within 0.05 of each other. The number of conformations occupying the same site was also considered and it was observed that the occupancy of the specific site of docking was at least over 50%. The best rank conformation of each of the three complexes are used to evaluate our experimental findings.

The theoretical ΔG^0^ values are in close agreement with the experimental values in all the cases [Table 2]. We find that AP (ΔG^0^ = −8.49 kcal mol^−1^) exhibits a higher affinity than BSA (ΔG^0^ = −7.58 kcal mol^−1^) and HSA (ΔG^0^ = −6.84 kcal mol^−1^).

**Figure 5:**
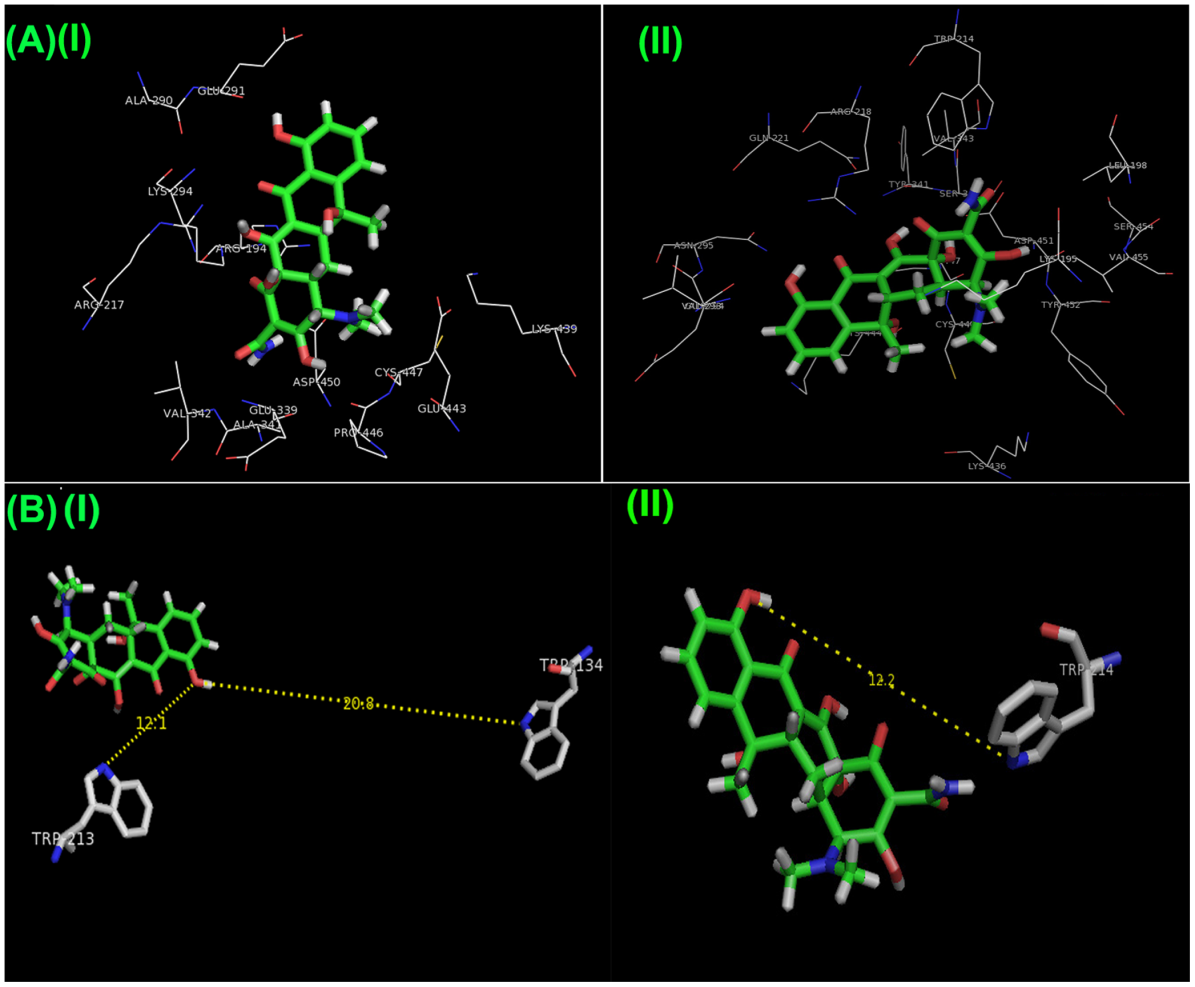
Docked poses of serum albumin-TC complexes. (A) The surrounding amino acid residues of (I) BSA (II) HSA within 5 Å from TC. (B) Distances (in Å) obtained from docked poses of different Trp residue/s of (I) BSA (II) HSA from TC.

The docked conformation of TC in the complexes serum albumin-TC are shown in Figure 5. Table 3 shows the different residues of serum albumins which lose over 10 Å^2^ in ASA upon binding with TC. Apart from these residues, Leu 198, Gln 221, Val 293, Tyr 341, Ser 342, Val 343, Lys 436, Ser 454 are also within 5 Å from the bound TC for the HSA-TC complex. Similarly, the residues Ala 290, Glu 339, Ala 341, Pro 446, Cys 447 are also found to be present within 5 Å from the bound TC for the BSA-TC complex. It is also noticed that the residues Lys 195, Arg 218, Gln 221, Glu 294, Asn 295, Ser 342, Lys 436, Lys 444, Cys 448, Asp 451, Tyr 452, Ser 454 make polar contacts with the probe TC in the HSA-TC complex. The residues Arg 194, Arg 217, Lys 294, Glu 339, Val 342, Asp 450 are found to make polar contacts with TC in the BSA-TC complex. Thus, it has been observed that the number of residues involved in binding TC is highest in the case of HSA-TC (Table 3, Figure 5) whereas in AP-TC only six residues are around the drug. [20] The docked pose indicates that TC binds in a buried region of HSA whereas the binding regions are partly buried and solvent exposed in the case of BSA and AP respectively. [20].

**Table 3:** The Changes in Accessible Surface Area (ΔASA)^a^ of the Residues of Serum Albumins after Complexed with Tetracycline.

HSA-TC		BSA-TC	
RESIDUE	ΔASA (Å^2^)	RESIDUE	ΔASA (Å^2^)
Lys195	−40.43	Arg 194	−25.87
Trp214	−27.02	Arg 217	−39.58
Arg218	−37.41	Glu 291	−39.5
Glu294	−24.71	Lys 294	−29.52
Asn295	−20.28	Val342	−18.45
Lys444	−12.48	Lys439	−15.50
Pro447	−17.38	Glu443	−14.16
Cys448	−34.80	Asp450	−39.41
Asp451	−30.12		
Tyr452	−18.98		
Val455	−11.22		
Total loss of ASA	−274.83	Total loss of ASA	−221.99

a ΔASA_i_ = ASA^i^_HSA/BSA_ – ASA^i^_HSA/BSA – TC complex._

The distances of Trp residues of the three proteins from TC and the changes in accessible surface area (ΔASA) of Trp residues are summarized in Table 4.

**Table 4:** Distances and of Tryptophan Residues of Different Proteins from TC Changes in Accessible Surface Area (ΔASA) of Tryptophan Obtained by the Docking Studies.

Distance (Å) from	BSA Trp 213	BSA Trp 134	HSA Trp 214	AP Trp 109	AP Trp 220	AP Trp 268
indole N to O attached with3-C	10.00	30.42	6.25	34.10	6.52	46.02
indole N to 5-C	10.74	26.46	8.6	30.67	5.56	44.8
indole N to O attached with10-C	12.13	20.84	12.21	24.23	7.57	40.23
indole N to 12-C	9.63	25.67	8.01	28.66	4.99	42.07
C_1_ to N attached with 4-C	13.76	30.99	10.15	35.41	9.00	47.58
C_1_ to O attached with 6-C	14.20	27.68	12.20	31.10	8.77	44.63
C_1_ to O attached with 11-C	11.30	24.72	10.50	36.08	8.56	46.82
ASA (Protein) (Å^2^)	40.9	15.4	61.7	1.0	52.7	46.7
ASA (complex)(Å^2^)	40.9	15.4	34.7	1.0	29.1	46.7
ΔASA (Å^2^)	0	0	27.0	0	23.6	0

### 3.4. Energy Transfer (FRET) in the Protein-TC Complexes

The steady-state and the time resolved quenching of serum albumins by TC are utilized to determine the energy transfer efficiency (E) in both the complexes of BSA and HSA using
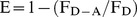
(10a)where, F_D_ and F_D-A_ are the fluorescence intensities of the proteins in the absence and in the presence of drug TC.

Since there is no overlap of the donor (Trp) emission and the emission of TC, E is also determined monitoring the enhancement of the emission of the acceptor TC using the following equation:
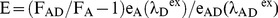
(10b)where, F_A_ and F_AD_ are the fluorescence intensities of the acceptor TC in the absence and in the presence of donor serum albumins with excitation at 280 nm. *ε*_A_ (*λ*_D_^ex^) and *ε*_AD_ (*λ*_AD_^ex^) are the extinction coefficient of the acceptor TC at 280 nm in the absence and in the presence of donor serum albumins. The energy transfer efficiencies for the serum albumins calculated by donor quenching and acceptor enhancement are given in Table 5. The greater value of energy transfer efficiency obtained (0.51 from donor quenching) in AP-TC complex [20] reflects that the probe molecule resides nearer to the Trp residue in AP than that in the case of serum albumins. Docking results discussed in section 3.3 also support this view.

**Table 5:** The Energy Transfer Efficiency and The Rate Constant of Protein-TC Complexes at 298 K.

Method	ET efficiency		Rate of ET (*k*_ET_)×10^8^				Ratio of (*k*_ET_) (HSA/BSA)		Theoritical Ratio of (*k*_ET_) (HSA/BSA)
			Eqn 11		Eqn 12		Eqn 11	Eqn 12	
	HSA	BSA	HSA	BSA	HSA	BSA			
i) Using Donor quenching	0.420.48 a	0.450.47 a	2.15	1.673	4.0	3.36	1.28	1.19	1.18^b^
ii) Using Acceptor enhancement	0.50	0.55	2.37	2.33	4.21	3.81	1.02	1.105	
iii) Using Phosphorescence quenching	0.33	0.35	1.14	1.01	0.76	0.66	1.13	1.15	

a Ref [66].

b Considering the distance from indole N of Trp to O attached with 10-C of TC.

The rate constant of the energy transfer (*k*_ET_) has been evaluated using
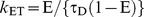
(11)and
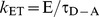
(12)where, τD and τD−A represents the average lifetime for the donor and the complex (after full complexation). The kET values thus obtained are provided in Table 5.

The rate constants k_ET_, in s^−1^, for energy exchange between two species coupled by the Förster transfer mechanism [68]–[69] is given by
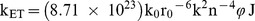
(13)where, k_0_ is the decay constant for the donor in the absence of the acceptor, φ is the radiative quantum yield in the absence of energy transfer, r is the distance between the centres of the donor and the acceptor (in Å), n is the refractive index of the medium, J is the overlap integral between the donor luminescence and the acceptor absorption spectrum, and κ^2^ is the orientation factor for the dipole-dipole interaction.

Assuming *J*, *n*, φ, and κ^2^ to be same for the systems HSA-TC and BSA-TC, one can calculate the ratio of the *k*_ET_ in the two systems using the donor-acceptor distance (*r*) obtained from the docking studies and the average lifetime of the donor (<τ_D_>).
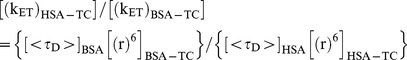
(14)


The theoretical ratio of *k*_ET_ for serum albumin-TC complexes using the distance between the indole N to O attached with 10-C of TC as donor-acceptor distance (Figure 5B) is presented in Table 5. The result calculated is compared with the experimental ratio of the *k*_ET._ Although the transition dipole moment direction of Trp is known, the direction of the transition dipole moment of TC is not known. Thus it is difficult to predict the actual values of orientation factor κ^2^ and the exact distance ‘r’ for the donor-acceptor pair in the complexes. Considering this limitation, a good correlation between the theoretical ratio and the experimental ratio of *k*_ET_ values supports the distances obtained from docking studies.

### 3.5 Phosphorescence Spectra: Role of Trp and Tyr Residues in the Interaction

**Figure 6:**
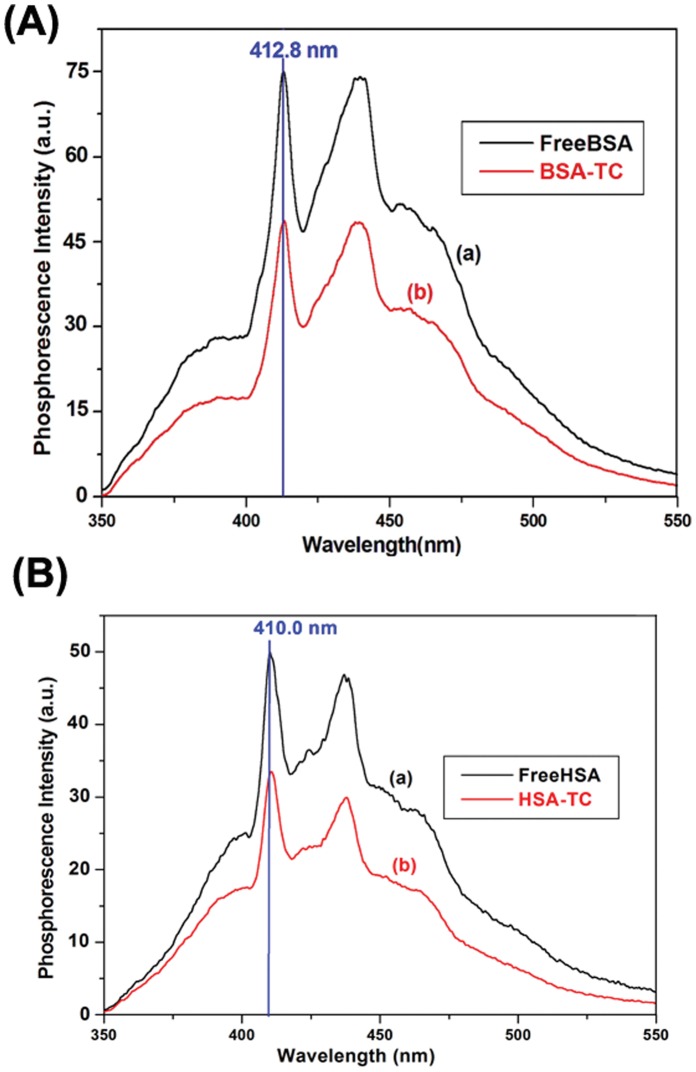
Phosphorescence spectra of (A) (a) 10 µM BSA and (b) BSA-TC (1∶1) complexes, (B)(a)10 µM HSA and (b) HSA-TC (1∶1) complexes in 40% EG matrix at 77 K; *λ*_exc_ = 280 nm. Excitation band pass = 10 nm and emission band pass = 1 nm for each case.

The phosphorescence spectra of free serum albumins and their complexes with TC in 40% EG glassy-matrix at 77 K are compared in Figure 6. The phosphorescence spectra in both the complexes are quenched compared with those of free proteins.

HSA contains a lone Trp residue at the position 214, which shows a phosphorescence (0,0) band at 410.0 nm. Upon binding to TC, there is a small but definitive red shift of the (0,0) band (Table 6). This indicates that Trp 214 experiences more hydrophobic environment upon binding. This result is also supported by the docking studies which indicates that a substantial loss of the ASA of Trp 214.

**Table 6:** Phosphorescence Data for Wild-Type Serum Albumins and its Complex with TC in a 40% Ethylene Glycol Matrix at 77 K (λ_exc_ = 280 nm).

System	Position of the phosphorescence (0,0) band (nm)	Width of the phosphorescence (0,0) band at half-maxima (cm^−1^)
Free BSA 10 µM)	412.8	270
BSA-TC (10∶10)	413.4	305
Free HSA 10 µM)	410.0	332
HSA-TC (10∶10)	410.6	320

Although BSA possesses two tryptophan residues, Trp 213 and Trp 134, the (0,0) band appears as a single peak at 412.8 nm and the two Trp residues cannot be optically resolved. However, BSA shows two distinct (0,0) bands upon binding with suitable fatty acid (406.1 nm and 414.1 nm), [70] 3-hydroxy Naphthoic Acid (405.6 nm and 412.8 nm), [43] catechin/epicatechin (403.8 nm and 412.0 nm) [71]. The blue shifted band is attributed to Trp 134 of BSA. In the BSA-TC complex, LTP shows only one (0,0) band at 413.4 nm indicating that Trp 134 is not at all perturbed in this interaction. The position of the (0,0) band for the BSA-TC complex is also slightly red shifted compared to that in free BSA. The slight difference in band widths of the (0,0) bands (Table 6) indicates that the Trp environments in free serum albumins and in their complexes are not similar in homogeneity.

The ET efficiency calculated from the Trp phosphorescence spectra of the free albumins and their complexes with *λ*_exc_ = 295 nm (where Tyr is not excited) is found to be 0.33 for HSA and 0.35 for BSA (Table 5) using equation:
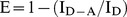
(15)where, I_D_ and I_D-A_ represent the area under the total phosphorescence spectra of the proteins in the absence and in the presence of drug TC respectively.

It is to be noted that although the non-radiative ET takes place at the singlet level, E values obtained from phosphorescence data at 77 K are in fairly good agreement with those calculated from the fluorescence data at room temperature (Table 5) and thus support our earlier conclusions. Quantitative agreement should not be expected, since ET may differ for fluid and rigid solvent media.

A similar study with AP showed that only Trp 220 among the three Trp residue is involved in the interaction with TC. [20] In the case of free serum albumins and their complexes with TC, an unstructured underlying emission originating near 350 nm was observed in the spectrum, which is assigned to Tyr residues. [41] For AP-TC complex the contribution of tyrosine phosphorescence appearing below 400 nm was found to be diminished considerably. [20] This result thus indicates that Tyr residues are not involved in the ET process in the interaction between serum albumins and TC.

### 3.6. Time Resolved Anisotropy of TC in the Complexes: Rotational Correlation Time

**Figure 7:**
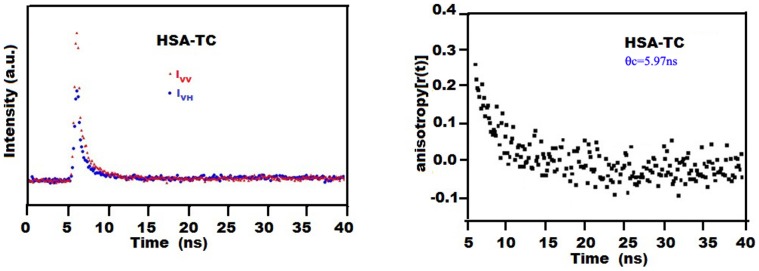
Fluorescence anisotropy decays of TC (25 µM) at 298 K in 25 µM has. I_VV_ and I_VH_ represent decays of emission of TC with excitation polarizer at vertical position and emission polarizer at vertical and horizontal position, respectively. λ_exc_ = 370 nm; excitation and emission band pass = 10 nm each.

The anisotropy decays of TC in the different complexes with proteins HSA (Figure 7) and BSA are measured using λ_exc_ = 370 nm and λ_exc_ = 405 nm. The rotational correlation times (θ_c_) are recovered from the fitted curves (Table 7). The results confirm that the enhancement of quantum yield of the probe TC in the complex is due to motional restriction imposed on the probe due to binding. The maximum value of θ_c_ (6 ns) observed for HSA–TC compared to that in BSA–TC (θ_c_ = 5 ns) and in AP-TC (θ_c_ = 3.6 ns) correlates well with the docking results where we find highest number of perturbed residues around the drug in HSA-TC complex (Table 3).The results also imply the buried nature of the binding site of TC in the case of HSA. This imposes restriction of rotational motion of TC leading to the enhanced value of θ_C_. The faster rotational correlation time of TC in AP-TC is in conformity with the solvent exposed nature of the drug chromophore. [20] BSA-TC complex exhibits intermediate value of θ_C_ confirming the partial exposed environment of TC (Table 3).

**Table 7:** Photophysical Data of TC Obtained by Monitoring the Emission Maxima in Different Medium and in the Complexes with Proteins at 298 K (λ_exc_ = 370 nm).

System (TC in)	λ_em_ (nm)	Φ (x10^3^)	Singlet state lifetime (ns)				Rotational correlation time θ_c_(ns)	(k_r_ x10^−6^) s^−1^	(k_nr_×10^−9^) s^−1^	Solvent Parrameters			
			τ_1_	τ_2_	τ_av_	χ^2^				π^*^	α	β	ε
DMSO	523.0	2.49	2.78 (18.24%)	1.53 (81.76%)	1.76	1.01	2.3	1.41	0.57	0.98	0.00	0.76	45.00
DMF	524.0	1.69	2.08 (9.81%)	0.91 (90.19%)	1.02	1.11	1.4	1.66	0.98	0.88	0.00	0.69	38.00
EG	530.0	1.44	1.93 (21.14%)	0.74 (78.86%)	0.99	0.99	3.4	1.46	1.01	0.88	0.90	0.52	37.00
EtOH	545.5	0.72	1.51 (13.78%)	0.58 (86.22%)	0.71	1.00	2.3	1.01	1.41	0.54	0.83	0.77	24.55
i-PrOH	548.0	0.52	1.87 (2.13%)	0.49 (97.87%)	0.52	1.20	3.0	1.00	0.92	0.48	0.76	0.95	18.00
Water	Broad ∼515	0.26	1.6 (5.06%)	0.3 (94.94%)	0.36	1.17	–	0.71	2.73	1.09	1.17	0.18	80.0
HSA	490.0	0.70	3.22 (11.2%)	0.96 (88.8%)	1.20	1.10	6.0 6.2^a^	0.59	0.83	–	–	–	–
BSA	498.0	1.55	2.62 (22.8%)	1.11 (77.2%)	1.45	0.99	5.0 4.9^a^	1.07	0.69	–	–	–	–
AP	509.0	2.30	2.79 (10%)	0.39 (90%)	0.63	0.90	3.6 3.5^a^	3.43	1.49	–	–	–	–

a The value obtained using λ_exc_ = 405 nm.

### 3.7 Steady State and Time Resolved Emission of TC in Various Solvents Exploring the Nature of the Microenvironments of Bound TC in the Complexes

**Figure 8:**
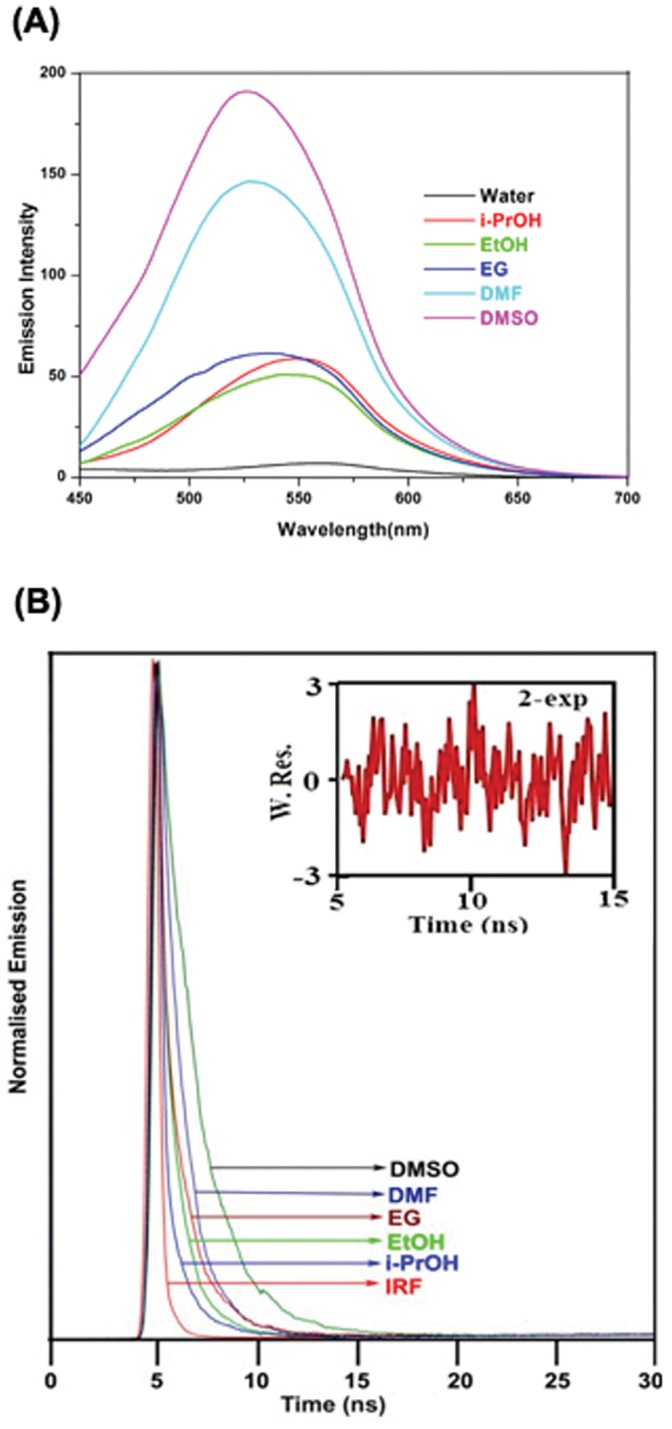
Steady State and Time Resolved Emission of TC in Various Solvents. (**A**) Fluorescence spectra of TC (25 µM) at 298 K in (1) water, (2) ethanol (EtOH), (3) isopropanol (iPrOH), (4) ethylene glycol (EG), (5) dimethylformamide (DMF), (6) dimethyl sulphoxide (DMSO); λ_exc_ = 370 nm; excitation and emission band pass = 10 nm and 5 nm respectively. (**B**) Fluorescence decay of TC (25 µM) at 298 K in (B) EtOH, iPr-OH, EG, DMF, DMSO; λexc = 370 nm; excitation and emission bandpass = 10 nm each.

**Figure 9:**
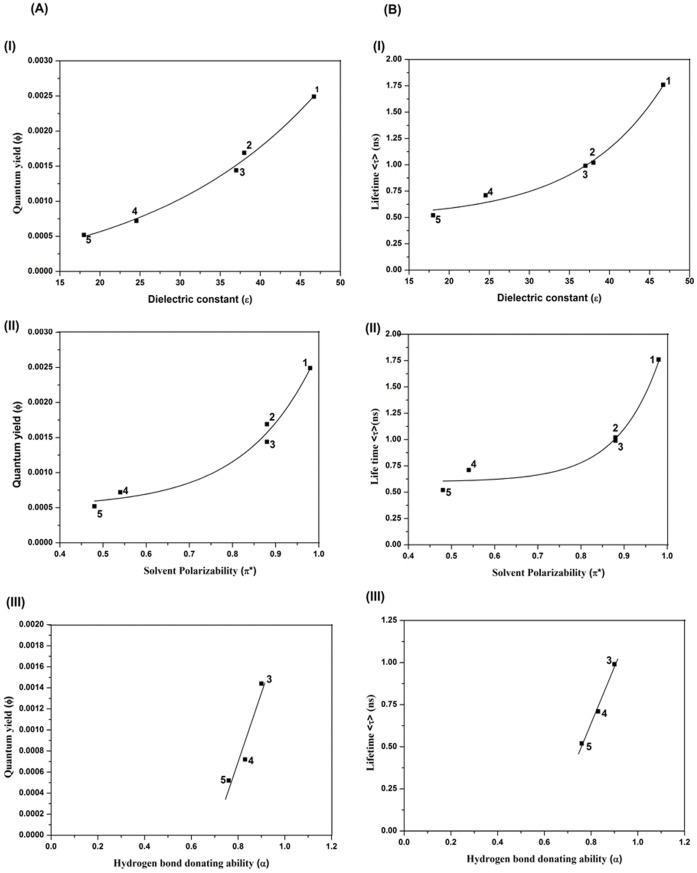
Plot of φ (A) and <τ> (B) against (I) dielectric constant (ε), (II) solvent polarizability parameter (π*), (III) Hydrogen bond donating ability (α) of different solvents, (1) DMSO, (2) DMF, (3) EG, (4) EtOH, (5) i-PrOH.

**Figure 10:**
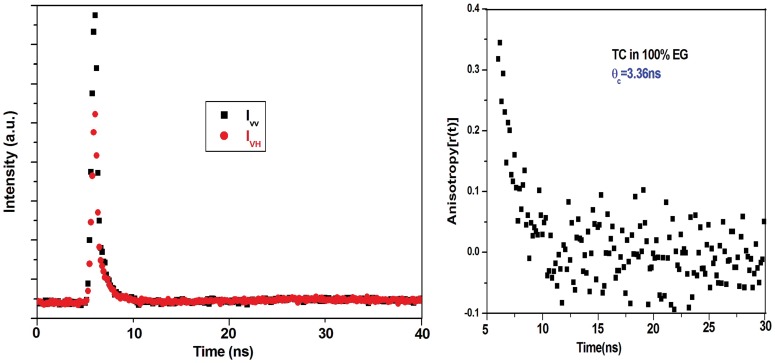
Fluorescence anisotropy decays of TC (25 µM) at 298 K in pure EG. I_VV_ and I_VH_ represent decays of emission of TC with excitation polarizer at vertical position and emission polarizer at vertical and horizontal position, respectively. λ_exc_ = 370 nm; excitation and emission band pass = 10 nm each.

In order to understand the details of the microenvironment of TC in all the protein-TC complexes we carried out the steady state and time resolved fluorescence of TC monitoring the λ_max_ of the emission of TC in various solvents as shown in Figure 8A and 8B respectively. The quantum yield (Φ) and the lifetime (τ) of the emission of TC in various polar protic and aprotic solvents have been provided in Table 7. The quantum yield and the singlet state lifetime are found to be higher in polar aprotic solvents compared to that in polar protic solvents (Table 7). The influence of solvent polarizability parameter π*, hydrogen bond donating ability (α) and hydrogen bond accepting ability (β) of different solvents on the emission of TC is studied using Kamlet–Taft multi-parameter approach. [72] In the plot of Φ vs dielectric constant (ε) and the average lifetime <τ> vs. ε, an increasing trend with increasing ε is observed (Figure 9). Plots of Φ and <τ> with π* show gradual enhancement of Φ and <τ> with increasing π*(Figure 9). The parameter β, however, does not exhibit any such correlation. For polar protic solvents, we observed a linear trend in the plot of Φ vs α and <τ> vs. α (Figure 9). These results imply that the polarity, the polarizability and the hydrogen bond donating ability of the surrounding residues control the emission quantum yield of TC in the protein-TC complex apart from the motional restriction of bound TC in the complexes. The radiative (k_r_) and the non-radiative (k_nr_) rate constants of the emission of TC in different solvents and in the complexes of proteins have been calculated (Table 7). The radiative rate constants vary for the different protein-drug complexes. It is found that k_r_ is maximum for AP-TC and minimum for HSA-TC complex (Table 7). In DMSO where the quantum yield is observed to be the highest, the nonradiative rate constant is found to be 0.57×10^−9^ s^−1^ compared to 2.73×10^−9^ s^−1^ in water (Table 7). Rotational correlation time measured from fluorescence anisotropy decay are given in Table 7. A representative decay in EG is shown in Figure 10. The rotational correlation time, θ_C_, is found to be maximum in EG among all the solvents used for study. Measurement of rotational correlation time of TC in aqueous buffer, however, is beyond our present detection limit.

### Conclusions

The steady state and time resolved studies monitoring the enhanced emission of bound TC at 298 K, the phosphorescence spectra of the free proteins and the protein-TC complexes at 77 K and the molecular docking studies are used to explore the interaction between the widely used antibiotic TC and proteins HSA, BSA. The results have been compared with those found for AP-TC complex. The studies reveal the following:

- The number of binding site is one for each protein and the binding constant at 298 K is of the order of ≈10^4^ for serum albumins, whereas for AP-TC it is two orders of magnitude greater.
- The TC emission is enhanced in the complex of proteins with φ_(AP-TC)_> φ_(BSA-TC)_ >φ_(HSA-TC)_. The experimental ratio of the ET rate constants [(k_ET_)_HSA-TC_/(k_ET_)_BSA-TC_] agree well with the ratio calculated using the distance and the orientation of the donor-acceptor obtained from the docking results. In the case of HSA and BSA, Trp 214 and Trp 213 are involved in the ET process respectively. The docking results and the phosphorescence spectra reveal that Trp 134 for BSA is not taking part in the ET process. The Tyr residues in both serum albumins are also found to be not involved in the interaction.
- Docking studies reveal that the number of residues in the binding region of TC and the total loss of ASA of different residues is maximum for HSA-TC complexes, whereas in the case of AP-TC the number of residues perturbed was found to be minimum. This observation indicates that TC is bound in a buried region in HSA, partly buried region for BSA and solvent exposed region in AP. This is also supported by the rotational correlation time of TC in different complexes ([θ_C_]_HSA-TC_ >[θ_C_]_BSA-TC_>[θ_C_]_AP-TC_).
- The emission maxima, the quantum yield, the lifetime and the rotational correlation time of the probe TC in various pure solvents imply that the polarity, the polarizability and the hydrogen bond donating ability of the neighbouring residues of TC along with the motional restriction of bound TC govern the enhanced emission of TC in different protein-TC complexes.
